# F12 as a reliable diagnostic and prognostic biomarker associated with immune infiltration in papillary thyroid cancer

**DOI:** 10.18632/aging.204037

**Published:** 2022-04-28

**Authors:** Jun-Hua Luo, Xiu-Xia Zhang, Wu-Hui Sun

**Affiliations:** 1 Department of Thyroid and Breast Surgery, Linping Campus, The Second Affiliated Hospital of Zhejiang University School of Medicine, Hangzhou 311100, Zhejiang, China; 2 Department of Thyroid Surgery, The Second Affiliated Hospital of Zhejiang University School of Medicine, Hangzhou 310009, Zhejiang, China

**Keywords:** coagulation factor XII, papillary thyroid cancer, biomarker, diagnosis, prognosis

## Abstract

Objective: To explore the function of coagulation factor XII (F12) in papillary thyroid cancer (PTC).

Materials and Methods: We assessed F12 expression and its relationship with overall survival (OS) in various cancers using TIMER and TISIDB databases. Further, we evaluated the mRNA and protein expression levels of F12 in PTC via different bioinformatics tools. The receiver operating characteristic (ROC) curve was applied to determine the diagnostic value of F12 in PTC. Then, the Kaplan-Meier plotter and Cox regression analyses were performed to examine the prognostic significance of F12. The possible mechanism of F12 in PTC was investigated through enrichment analyses. Finally, the correlation between F12 expression and immune cell infiltration was analyzed using TCGA data.

Results: This study revealed the clinical significance of F12 in various cancers. Higher mRNA (P <0.001) and protein expressions of F12 were observed in PTC compared with normal tissues. Besides, F12 expression exhibited high diagnostic performance in PTC and its overexpression served as an independent predictor for the poor OS (P <0.05). Enrichment analyses results showed that F12 was mainly involved in metabolism-associated pathways. Additionally, F12 expression was significantly linked to immune cell infiltration levels, especially macrophage infiltration.

Conclusions: F12 might be a reliable diagnostic and prognostic biomarker for PTC. Moreover, F12 expression might affect the OS of PTC patients via regulating metabolic pathways.

## INTRODUCTION

Thyroid cancer is one of the most common endocrine malignancies with increasing morbidity [1]. It is estimated that there were 95,030 newly diagnosed cases of thyroid cancer and 22,070 deaths in 1990, which increased to 255,490 new cases and 41,240 deaths in 2017 [2]. Papillary thyroid cancer (PTC) is the most predominant subtype of thyroid cancer, accounting for approximately 85% of all cases [3]. Radical surgery, thyroid-stimulating hormone suppression, and ^131^I therapy are the traditional approaches for the PTC treatment, displaying relatively satisfactory efficacy [4]. Although the 5-year survival rate of PTC patients is greater than 97% and the 10-year survival rate of such patients reached 85%, recurrence was found in roughly 15% and 28% of the patients within 10 years and 27 years, respectively after the initial treatment [5–8]. The majority of patients with PTC possess indolent progression, while overdiagnosis and overtreatment are common problems related to indolent diseases, which may increase the risk of injury to patients [9, 10]. Some biomarkers such as HBME-1 and thyroglobulin have been used for the diagnosis of PTC in clinical practice, whereas, these biomarkers presented low specificity and sensitivity [11, 12]. Therefore, it is imperative to develop more effective and sensitive biomarkers for improving the diagnosis and prognosis of PTC.

Coagulation factor XII (F12) also called the Hageman factor, is a single-chain zymogen with a molecular weight of about 80kDa [13]. It is produced by hepatocytes in the liver and consists of a heavy chain (353 residues) and a light chain (243 residues) [14, 15]. F12 is a circulating serine protease, which is activated as F12a by plasma kallikrein and negatively charged surfaces [16, 17]. The contact system driven by F12 can be both prothrombotic via activation of F11 to trigger the intrinsic pathway of coagulation, and be proinflammatory by activating the kallikrein-kinin pathway to release the peptide bradykinin [18]. The binding of bradykinin to the G-protein-coupled kinin B2 receptor initiates intracellular signaling pathways that may accelerate angiogenesis and cancer growth [17, 19]. F12 has been demonstrated to be involved in various diseases. It colocalized with Aβ plaques in the brain of patients with Alzheimer’s disease and in brain lesions of those with multiple sclerosis [20, 21]. F12 was also found to participate in stroke development and the inhibition of F12 might offer a selective strategy to prevent this disease [22]. In addition, low F12 plasma levels were observed in patients with colorectal, gastrointestinal, and lung cancer [23–25]. However, little research has been done on the role of F12 in PTC.

Herein, we adopted TIMER and TISISDB databases to analyze the clinical significance of F12 in pan-cancer. Then, the expression analysis of F12 in PTC was performed through multiple bioinformatics tools. The correlation between F12 expression and PTC patient prognosis was analyzed using the TCGA data. The enrichment analyses were applied to reveal the potential molecular mechanism for F12 in PTC. Moreover, we assessed the association of F12 expression with immune cell infiltration levels. This study identified the potential role of F12 in PTC occurrence and progression and provided that F12 might be a reliable therapeutic target for PTC treatment.

## MATERIALS AND METHODS

### Expression and survival analyses of F12 in pan-cancer

TIMER (https://cistrome.shinyapps.io/timer/) is a comprehensive resource for the systematic analysis of immune infiltrates across diverse cancer types. The database was used to analyze the expression levels of F12 in different kinds of tumors with Wilcoxon tests. Next, the associations between F12 expression and overall survival (OS) across human cancers were assessed using the TISIDB (http://cis.hku.hk/TISIDB/), which is an integrated repository portal for tumor-immune system interactions.

### The genetic status and expression level of F12 in PTC

Firstly, the cBioportal database (https://www.cbioportal.org/) was adopted to obtain the genetic alteration information by selecting “Papillary Thyroid Carcinoma (TCGA, Cell 2014)” with 496 total samples and entering “F12” for the query. Then, gene expression profiles and relevant clinical data of GDC TCGA-thyroid cancer were retrieved from the UCSC Xena (https://xenabrowser.net/). Totally 498 PTC samples with complete survival and expression data were enrolled for further analyses. In this dataset, the statistical significance of the F12 expression levels was evaluated in PTC tissues and adjacent normal tissues using unpaired and paired t-tests*.* The immunohistochemical images of F12 protein in PTC and healthy thyroid tissues were obtained from the HPA (https://www.proteinatlas.org/) database. The protein expression is scored with regard to staining intensity (strong, moderate, weak, or negative) and the fraction of stained cells (>75%, 25-75%, or <25%). Subsequently, all the included patients were divided into two groups according to the median expression of F12. The relationship between F12 expression and clinicopathological characteristics was analyzed using Pearson’s chi-square test.

### Survival analysis

The diagnostic value of F12 in PTC was initially analyzed by calculating the area under the curve (AUC) of the receiver operating characteristic (ROC) curves. Using the “maxstat” packages in the R program, the 498 PTC patients were divided into high- and low- F12 expression groups by setting the best cutoff value as the parameter. Then, F12 mRNA expression and different population OS curves were plotted by the Kaplan-Meier method using “survival” packages in the R program. Log-rank tests were employed to evaluate the survival differences between two expression groups. The ROC curve analyses were also conducted to determine the value of F12 in predicting the survival status of the PTC patients. Moreover, univariate and multivariate Cox regression analyses were performed to assess the correlation between OS and clinical pathological factors in PTC patients. Male was a reference level for gender, while tumor-free for cancer status, and stage 1 for the stage.

### Identification of F12-related genes and enrichment analyses

Based on the median value of F12 expression, 498 PTC patients were classified into high- and low- F12 expression groups. Then, the “limma” package in R was used to identify the differentially expressed genes (DEGs) between the two expression groups. Genes with |log2 foldchange (FC)|>1 and P <0.05 were considered statistically significant for the DEGs. A PPI network of the top 200 DEGs was constructed in the STRING database (https://cn.string-db.org/) and subsequently was visualized by Cytoscape software. After that, the top 200 DEGs were subjected to gene ontology (GO) annotations and Kyoto Encyclopedia of Genes and Genomes pathway analyses using the R package “clusterProfile”. The GO annotations included biological process (BP), cellular component (CC), and molecular function (MF). The cutoff value for significant function and pathway screening was set at P <0.05 and false discovery rate (FDR) <0.25.

Further, 498 PTC samples were divided into high and low expression groups using F12 expression median level as a cutoff criterion. The gene set enrichment analysis (GSEA) was performed to illustrate the significant survival difference between the two expression groups. The gene set was permutated 1000 times and the expression level of F12 was used as a phenotypic label. A nominal p-value <0.05 and an FDR q-value <0.25 were considered to be statistically significant.

### Immune cell infiltration analysis

The MCP-counter algorithm was employed to estimate the correlation of eight immune cells and two stromal cells’ infiltration levels with F12 mRNA expression. In addition, the immune response of 22 tumor-infiltrating immune cells was measured to assess their association with F12 mRNA expression by the CIBERSORT which is a deconvolution algorithm based on gene expression [26]. Samples from TCGA were divided into high F12 (50%) and low F12 (50%) expression groups to compare the level of immune cell infiltration.

### Statistical analysis

All statistical analyses were performed in SPSS software (version 23.0) and packages of R (version 3.6.3). Image J software was used to quantify the F12 protein content in PTC and normal thyroid tissues. Pearson’s correlation test was employed to evaluate the association of F12 expression with the immune cell infiltration levels and the expression of the markers of macrophages. P <0. 05 was considered to be statistically significant.

## RESULTS

### The clinical value of F12 in pan-cancer

**Figure 1:**
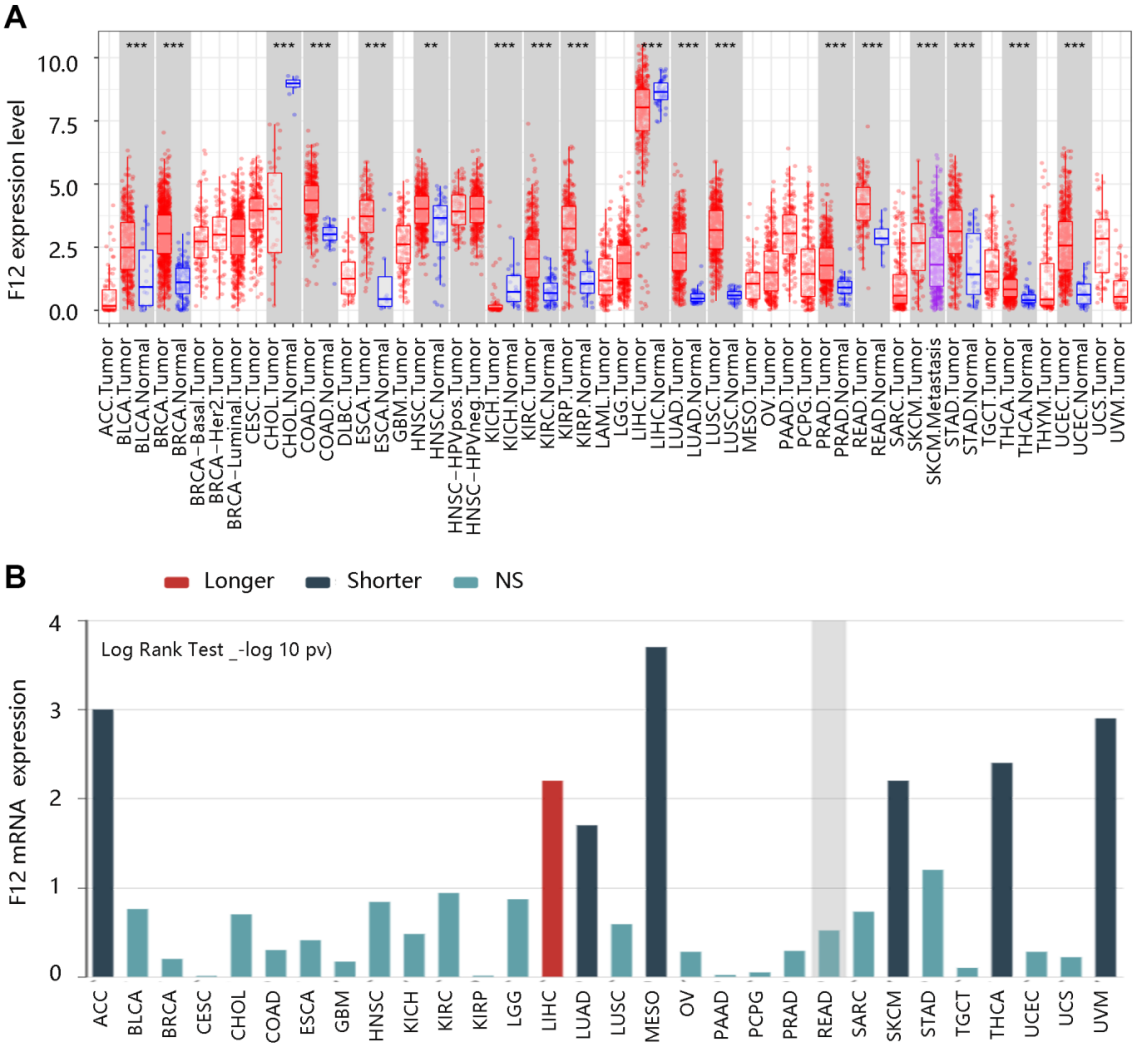
**The clinical value of F12 in pan-cancer.** (**A**) The expression analysis of F12 in various cancers via TIMER. **P <0.01; ***P <0.001. (**B**) The survival analysis F12 across human cancers via TISIDB.

To identify the role of F12 in pan-cancer, the TIMER database was used to evaluate the expression levels of F12 in various kinds of cancers. Higher F12 expression was observed in most cancers including BLCA, BRCA, COAD, ESCA, HNSC, KIRC, KIRP, LUAD, LUSC, PRAD, READ, SKCM, STAD, THCA, and UCEC (all P <0.01) (Figure 1A). Then, we evaluated the association of F12 expression with OS across human cancers through the TISIDB database. As shown in Figure 1B, the increased expression of F12 led to a shorter OS time of patients with ACC, LUAD, MESO, SKCM, THCA, and UVM; however, LIHC patients with higher F12 expression had a favorable prognosis. These results indicated that F12 might play an important role in the occurrence and progression of various tumors including thyroid cancer.

### Genetic status and expression level of F12 in PTC

**Figure 2:**
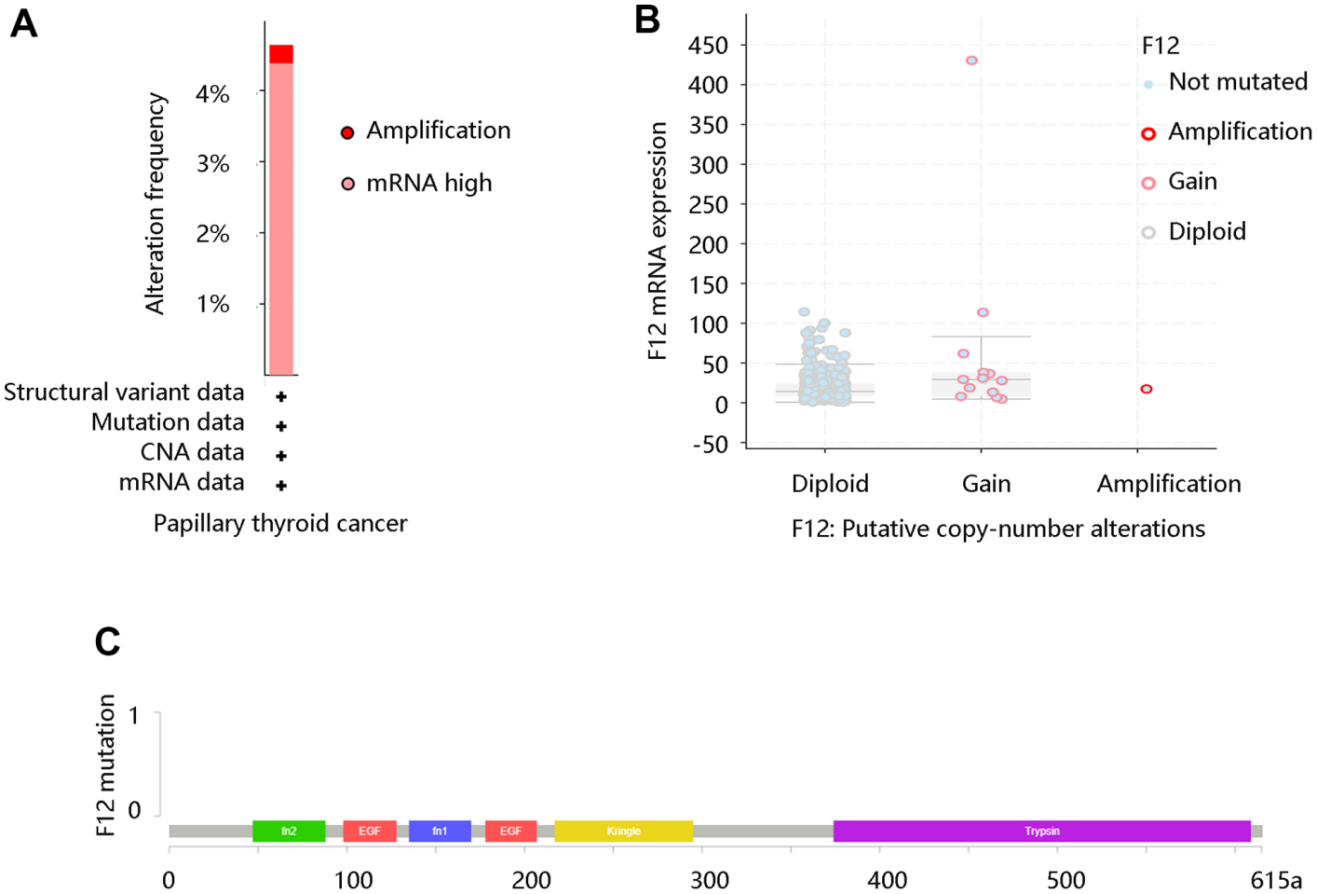
**The genetic status of F12 in PTC.** (**A**) The alteration frequency of F12 in PTC. (**B**) The copy number alteration of F12 in PTC. (**C**) The F12 mutation in PTC. PTC, papillary thyroid cancer.

Due to its aberrant expression in thyroid cancer, we analyzed its possible change from the aspect of genetic alterations. We firstly retrieved the alteration frequency of F12 in PTC using the cBioportal website. As exhibited in Figure 2A, mRNA high and amplification were major status. By analyzing the copy number of F12 gene in PTC, diploid and gain were primary status, indicating the elevated levels of F12 (Figure 2B). But surprisingly, F12 was not mutated in PTC (Figure 2C).

**Figure 3:**
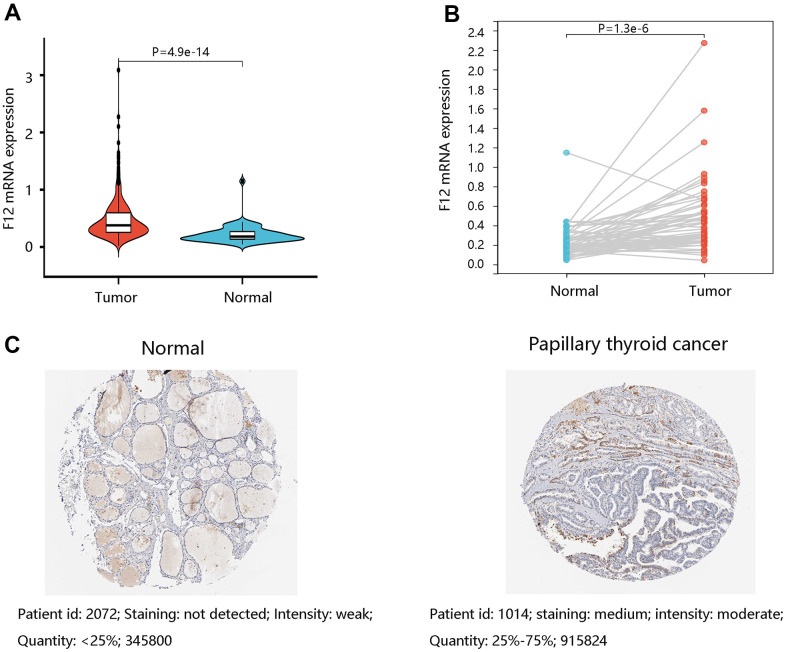
**The expression levels of F12 in PTC.** (**A**) The F12 mRNA expression in PTC and normal thyroid tissues. (**B**) The F12 mRNA expression in paired PTC samples. (**C**) The F12 protein expression in normal thyroid and PTC tissues. Image J software was used to quantify the protein levels of F12. PTC, papillary thyroid cancer.

Moreover, the TCGA data were applied to assess the F12 mRNA expression levels in PTC. The unpaired t-test (P =4.9e-14) (Figure 3A) and paired t-test (P =1.3e-6) (Figure 3B) presented higher F12 expression in PTC tissues than that in normal tissues. Following this, the protein levels of F12 were determined by the HPA database and the amount of F12 proteins was quantified by Image J software. As expected, higher F12 protein expression was observed in PTC tissue compared with the normal thyroid tissue. The staining was not detected and the intensity was weak in normal thyroid tissue, while medium staining and moderate intensity were found in PTC tissue (Figure 3C). These findings suggested that F12 overexpression might be essential in PTC tumorigenesis.

After characterizing the remarkable differential expression of F12 in PTC and normal thyroid tissues, we evaluated the clinical factors affecting its expression. As shown in Table 1, age, gender, cancer status, and stage were all not significantly linked to F12 mRNA expression (all P >0.05).

**Table 1:** Relationship between F12 mRNA expression and clinicopathological characteristics in papillary thyroid cancer.

**Characteristics**	**F12 low (%)**	**F12 high (%)**	**χ*^2^***	**P-value**
Age			2.946	0.086
<55	176 (52.7)	158 (47.3)		
≥55	73 (44.5)	91 (55.5)		
Gender			0.041	0.839
Female	184 (50.3)	182 (49.7)		
Male	65 (49.2)	67 (50.8)		
Cancer status			0.001	0.980
Tumor free	200 (49.8)	202 (50.2)		
With tumor	14 (50)	14 (50)		
Stage			4.618	0.202
Stage 1	153 (54.3)	129 (45.7)		
Stage 2	22 (43.1)	29 (56.9)		
Stage 3	49 (44.5)	61 (55.5)		
Stage 4	24 (46.2)	28 (53.8)		

### F12 had a high diagnostic efficiency

**Figure 4:**
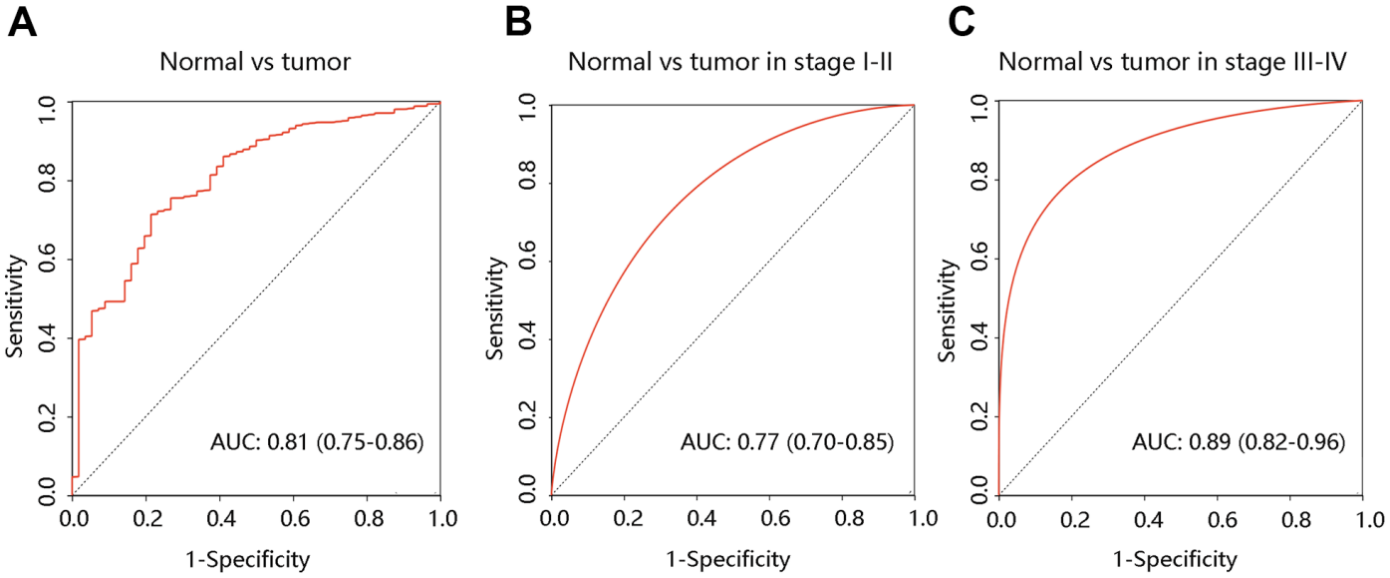
**Diagnostic value of F12 in PTC.** The ROC curves of F12 expression in PTC vs normal tissues (**A**) and different clinical stages (**B**, **C**). PTC, papillary thyroid cancer; ROC, receiver operating characteristic; AUC, the area under the curve.

To determine the value of F12 in diagnosis, the ROC curves were generated using the TCGA data. The entire AUC for F12 was 0.81 (95% confidence interval [95% CI]: 0.75-0.86), implying that F12 was capable of distinguishing normal individuals from PTC patients (Figure 4A). Besides, subgroup analysis demonstrated that the AUC values for stage I-II and stage III-IV groups were 0.77 and 0.89, respectively (Figure 4B, 4C). These results suggested that F12 had reliable diagnostic efficiency in discriminating the PTC patients and healthy subjects.

### High F12 expression predicted poor OS in PTC

**Figure 5:**
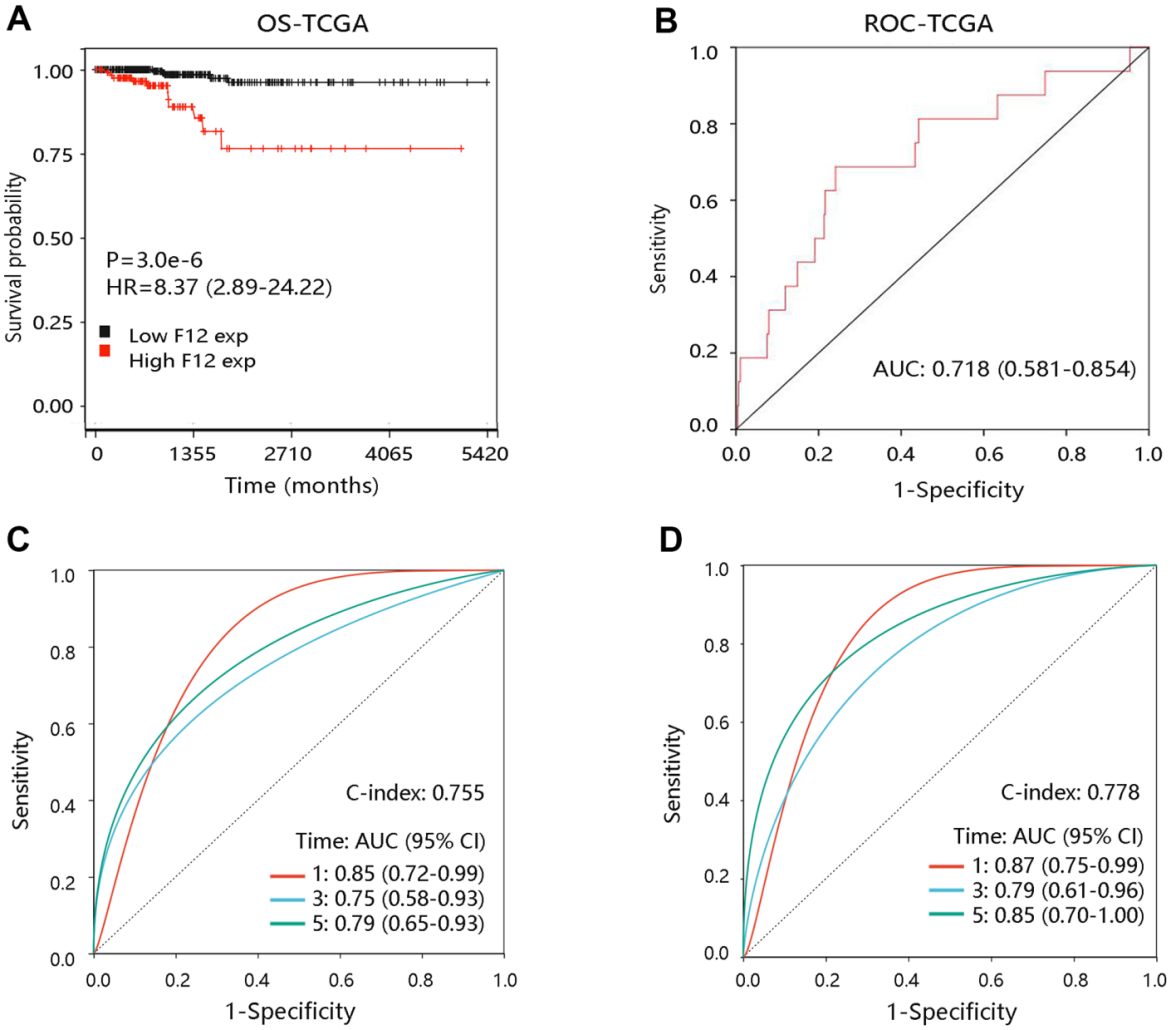
**The prognostic value of F12 in PTC.** (**A**) The association of F12 expression with OS of PTC patients. (**B**) The ROC curve of F12 expression for predicting the survival status. (**C**) Time-dependent ROC analysis in all PTC patients. (**D**) Time-dependent ROC analysis in PTC patients at stage III-IV. PTC, papillary thyroid cancer; ROC, receiver operating characteristic; OS, overall survival; HR, hazard ratio; AUC, area under the curve.

To explore the effect of F12 on OS of PTC patients, survival curves were drawn using the Kaplan-Meier method. Notably, PTC patients with higher F12 expression had worse OS than those with low F12 expression (P <0.001) (Figure 5A). The ROC curve result showed that the entire AUC was 0.718 (95% CI: 0.581-0.854) (Figure 5B). After considering the time factor, we found that F12 still had satisfactory performance in predicting OS status of PTC patients (concordance index [C-index]: 0.755; the AUCs of 1-, 3-, and 5-year were 0.85, 0.75, and 0.79, respectively) (Figure 5C). Time-dependent ROC analysis in stage III-IV PTC patients showed that the AUCs were 0.87, 0.79, and 0.85 for 1-, 3-, and 5-year, separately (C-index: 0.778) (Figure 5D). The time-dependent ROC analysis was not performed in patients at stage I-II due to fewer death samples. These findings revealed the potential value of F12 in predicting the survival status of PTC patients.

**Figure 6:**
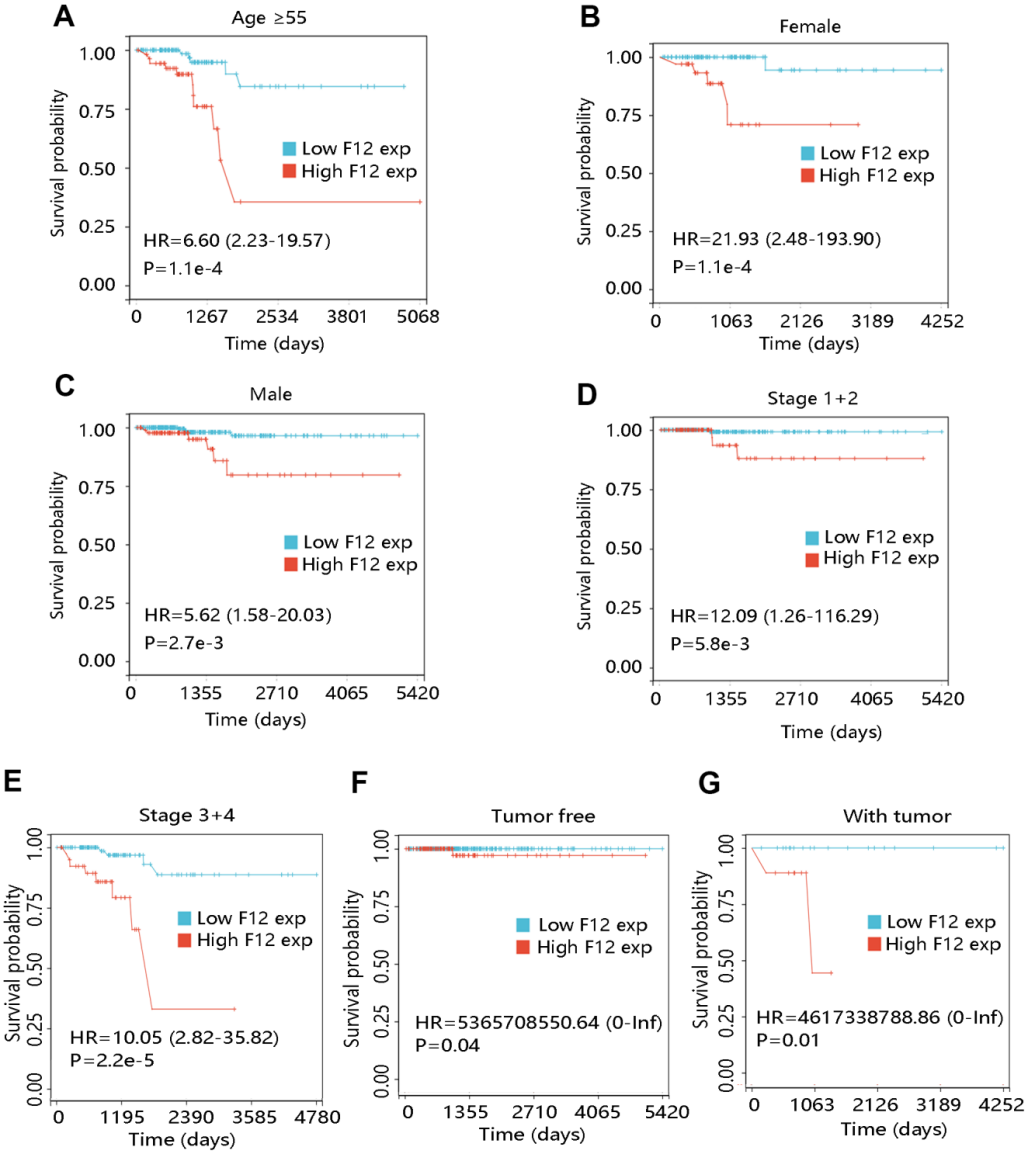
**Effect of F12 expression on overall survival of papillary thyroid cancer patients with restricted clinicopathological characteristics.** (**A**) Age ≥55. (**B**) Female. (**C**) Male. (**D**) Stage 1+2. (**E**) Stage 3+4. (**F**) Tumor free. (**G**) With tumor.

To further elucidate the effect of F12 overexpression on patient OS, we performed survival analyses on PTC patients with restricted clinicopathological characteristics. Due to no death occurring in patients less than 55 years, the survival analysis was not carried out in this subgroup. The elevated expression level of F12 had a significant relation with unfavorable OS in patients at age ≥55 years (P <0.001) (Figure 6A). In addition, high F12 expression led to the poor OS in subgroups of females, males, patients at stage 1+2, and stage 3+4 (all P <0.01) (Figure 6B–6E). F12 expression had a significant impact on the OS of PTC patients regardless of cancer status (all P <0.05) (Figure 6F, 6G).

To determine the independent prognostic value of F12 in PTC, Cox regression analyses were performed using the TCGA data. The univariate Cox regression analysis revealed the OS-related variables including age (Hazard ratio [HR] =1.160; P <0.001), cancer status (HR =30.615, P =0.005), stage 3 (HR =10.050, P =0.004), stage 4 (HR =20.622, P <0.001), and F12 (HR =4.894, P <0.001), while gender (P =0.180) and stage 2 (P =0.070) had no relationship with OS (Table 2). In the multivariate Cox regression analysis, age (HR =1.134, P =0.004) and F12 (HR =39.477, P =0.035) still had remarkable relationship with OS of PTC patients (Table 3). Therefore, high expression of F12 could independently predict poorer OS among PTC patients.

**Table 2:** Univariate Cox regression analysis of overall survival in papillary thyroid cancer patients.

**Characteristics**	**HR**	**95% CI-l**	**95% CI-u**	**P-value**
Age	1.160	1.101	1.222	<0.001
Gender	2.005	0.725	5.543	0.180
Cancer status	30.615	2.775	337.813	0.005
Stage 2	6.137	0.864	43.582	0.070
Stage 3	10.050	2.085	48.441	0.004
Stage 4	20.622	3.951	107.644	<0.001
F12	4.894	2.346	10.208	<0.001

Abbreviations: HR, hazard ratio; 95% CI-l, 95% confidence interval-lower; 95% CI-u, 95% confidence interval-upper.

**Table 3:** Multivariate Cox regression analysis of overall survival in papillary thyroid cancer patients.

**Characteristics**	**HR**	**95% CI-l**	**95% CI-u**	**P-value**
Age	1.134	1.041	1.235	0.004
Gender	0.087	0.000	74.469	0.479
Cancer status	1.961	0.000	3038836.204	0.926
Stage 2	0.000	0.000	1.218E+233	0.962
Stage 3	0.001	0.000	10242.999	0.395
Stage 4	0.000	0.000	2.962E+28	0.825
F12	39.477	1.305	1194.594	0.035

Abbreviations: HR, hazard ratio; 95% CI-l, 95% confidence interval-lower; 95% CI-u, 95% confidence interval-upper.

### Identification of F12-related genes and enrichment analyses

**Figure 7:**
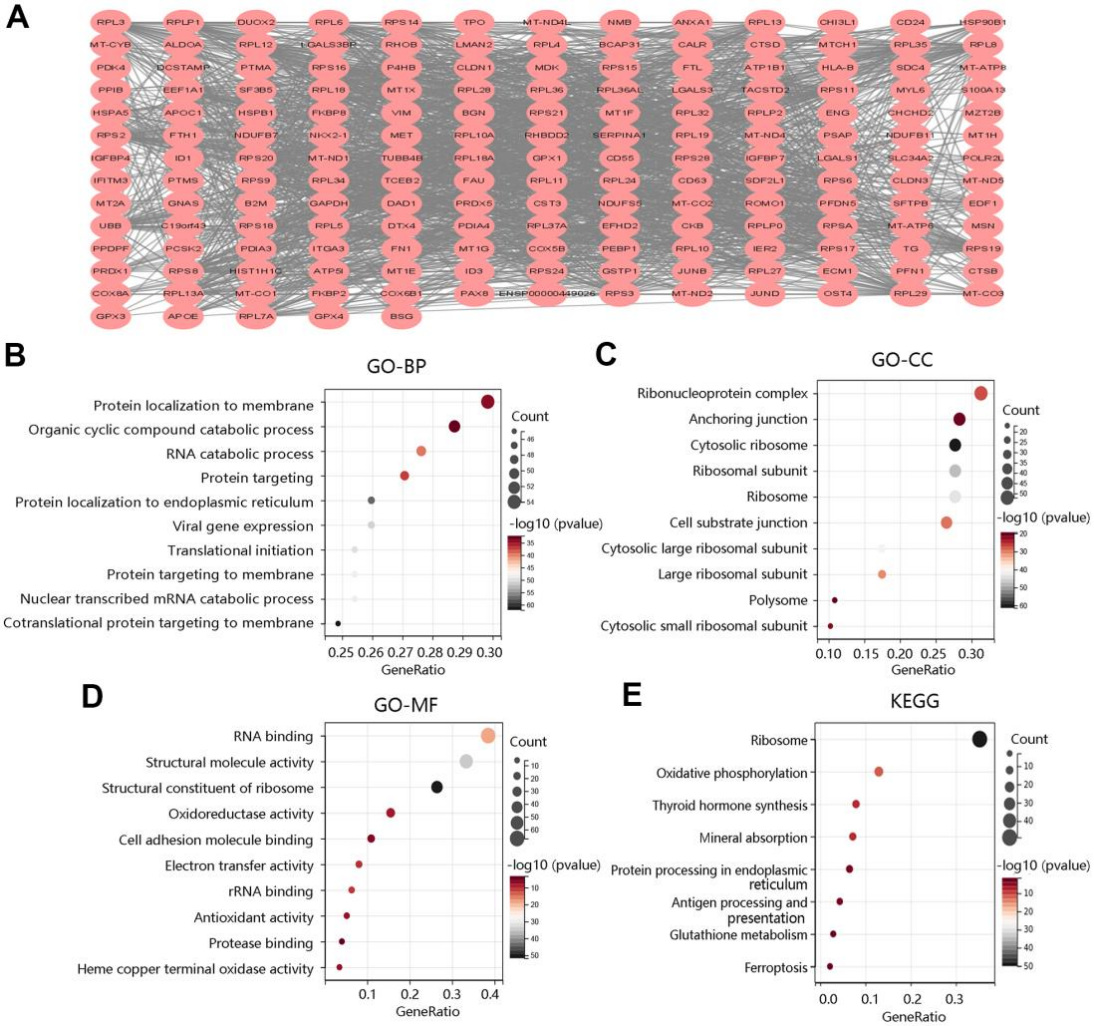
**Functional enrichment analysis of top 200 DEGs between high and low F12 expression groups.** (**A**) Protein-protein interaction network of top 200 DEGs. Gene ontology annotations of (**B**) biological process, (**C**) cellular component, and (**D**) molecular function. (**E**) KEGG pathway. DEGs, differentially expressed genes.

To explore the biological functions of F12 in PTC, we obtained the DEGs between high- and low- F12 expression groups for functional enrichment analysis. According to the selection criterion, the top 200 DEGs were selected for analysis, and the PPI network of these 200 DEGs was constructed using the STRING database, visualized by Cytoscape software (Figure 7A). Then, the selected genes were used for GO annotation and KEGG pathway analyses. As for BP, the genes were mainly enriched in protein localization to membrane, organic cyclic compound catabolic process, and RNA catabolic process (Figure 7B). For CC, they were mainly involved in ribonucleoprotein complex, anchoring junction, and ribosomal subunit (Figure 7C). The major MFs were RNA binding, structural; molecule activity, and structural constituent of ribosome (Figure 7D). The KEGG pathways that they participated in were oxidative phosphorylation, thyroid hormone synthesis, antigen processing and presentation, glutathione metabolism, and ferroptosis, which were closely related to tumor progression (Figure 7E).

**Figure 8:**
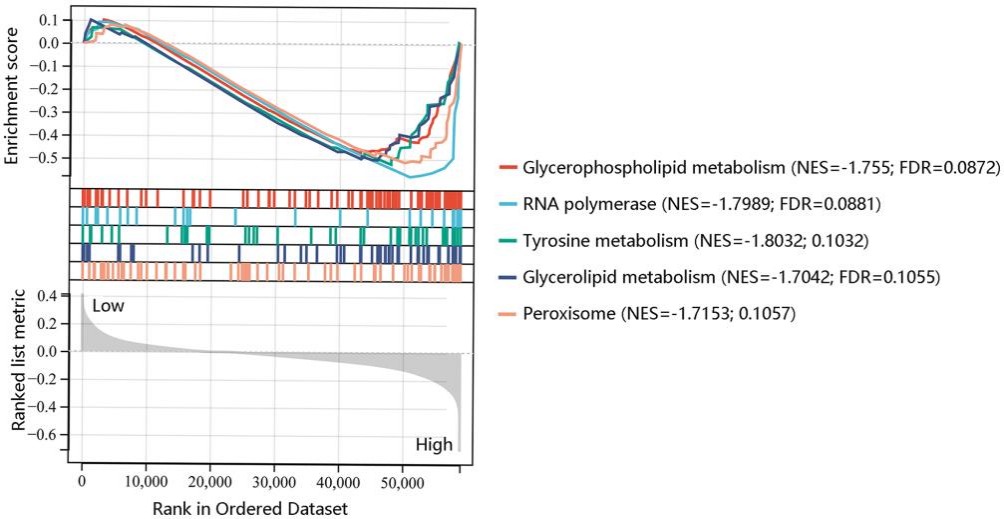
Gene set enrichment analysis of the five most significant pathways in high F12 expression phenotype.

To further reveal the underlying mechanism of F12 involved in PTC, GSEA was performed to exhibit the significantly enriched KEGG pathways in high F12 expression phenotype. The top 5 significant pathways were glyceropholipid metabolism (NES=-1.755, FDR=0.0872), RNA polymerase (NES=-1.7989, FDR=0.0881), tyrosine metabolism (NES=-1.8032, FDR=0.1032), glycerolipid metabolism (NES=-1.7042, FDR=0.1055), and peroxisome (NES=-1.7153, FDR=0.1057) (Figure 8). Thus, F12 might participate in the development of PTC through the regulation of these metabolic pathways.

### F12 expression was associated with immune cell infiltration

**Figure 9:**
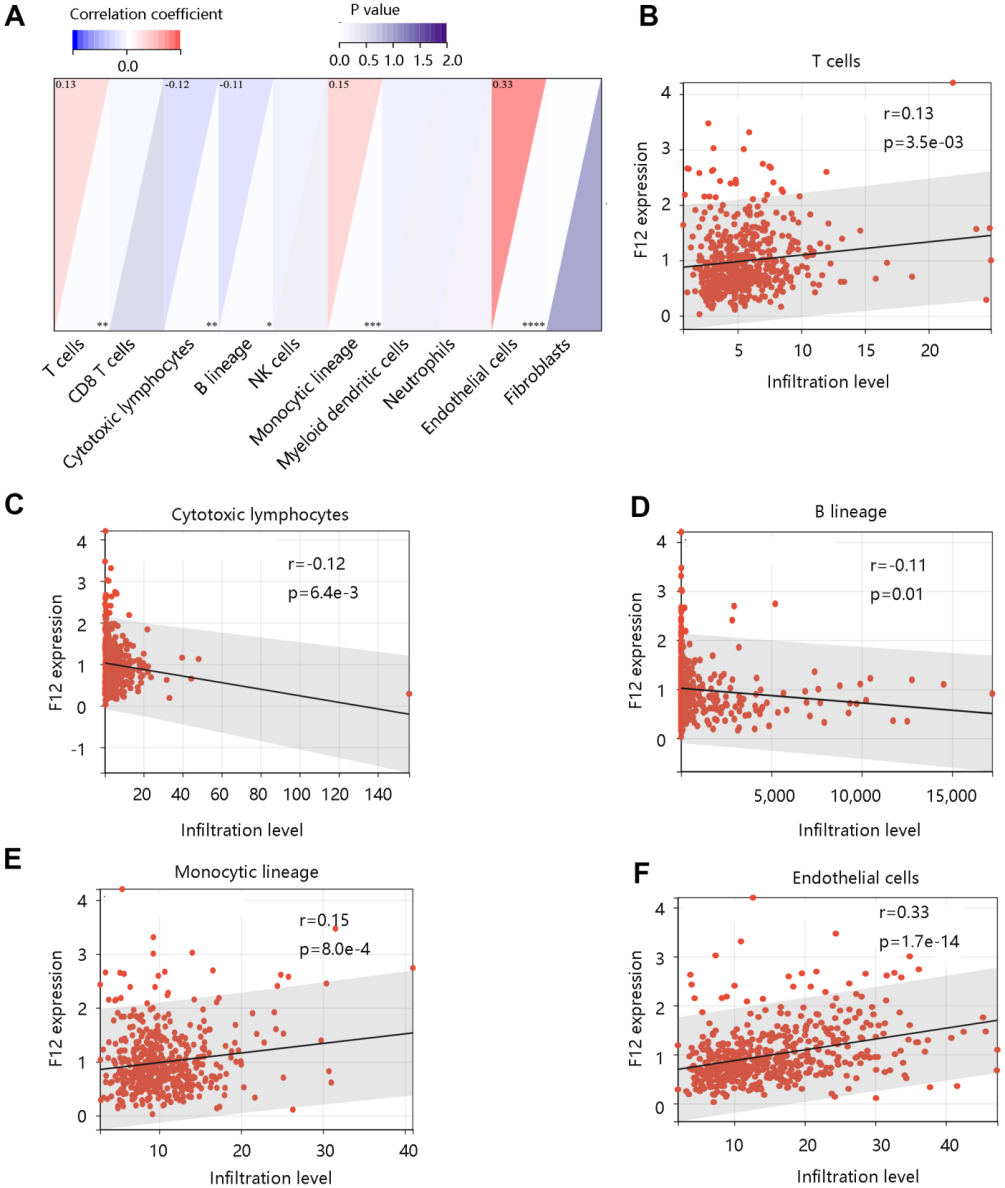
**Correlation between F12 and immune cell infiltration in papillary thyroid cancer by MCP-counter.** (**A**) The association of F12 with immune and stromal cell infiltration level. The significant relationship between F12 expression and (**B**) T cells, (**C**) cytotoxic lymphocytes, (**D**) B lineage, (**E**) monocytic lineage, and (**F**) endothelial cells.

**Figure 10:**
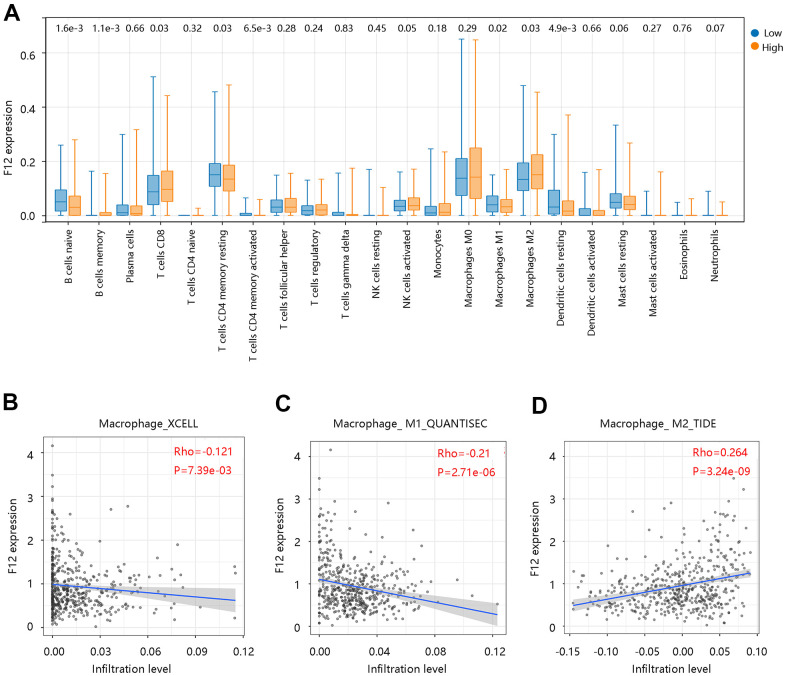
**Correlation between F12 and immune cell infiltration in papillary thyroid cancer by CIBERSORT.** (**A**) Immune cell infiltration level in the low- and high- F12 expression group. (**B**) Association of F12 expression with macrophage infiltration levels. (**C**) Association of F12 expression with M1 macrophage infiltration level. (**D**) Association of F12 expression with M2 macrophage infiltration level.

To assess the possible effect of F12 on various immune cell types in the PTC microenvironment, we first adopted the MCP-counter to assess the association of eight immune cells and two stromal cells’ infiltration levels with the F12 mRNA expression in PTC (Figure 9A). The F12 mRNA expression was significantly related to T cells, cytotoxic lymphocytes, B lineage, monocytic lineage, and endothelial cells (all P <0.05) (Figure 9B–9F). Following this, the CIBERSORT was used to calculate the fractions of 22 immune cells between high and low F12 expression groups in PTC samples. As shown in Figure 10A, the fractions of B cells naïve, B cells memory, T cells CD8, T cells CD4 memory resting, T cells CD4 memory activated, macrophages M1, macrophages M2, dendritic cells resting in low F12 expression group were significantly different from the high F12 expression group (all P <0.05). Interestingly, the M1 macrophage infiltration level was low, while that of M2 macrophages was high in the high F12 expression group. This suggested that high expression of F12 might promote the polarization of macrophages, which was intimately associated with the immunosuppressive state of the tumor [27]. Moreover, the TIMER web tool was used to validate the relationship between F12 expression and macrophage infiltration level with different algorithms. Figure 10B showed that F12 was negatively correlated with macrophage infiltration level (P =7.39e-03). Besides, F12 expression was negatively related to the M1 infiltration level but positively linked to the M2 infiltration level (all P <0.001) (Figure 10C, 10D).

**Figure 11:**
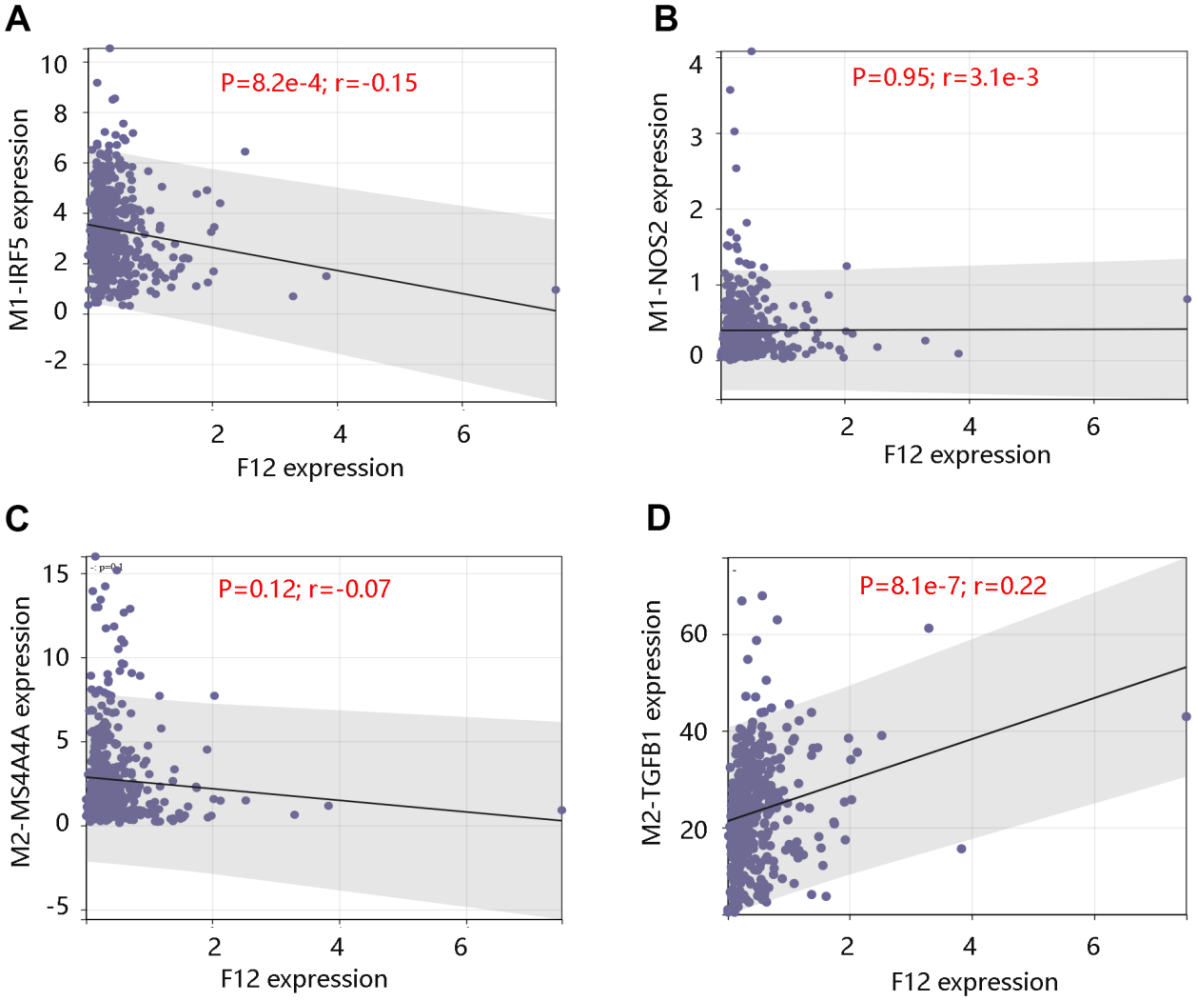
**Correlation between F12 expression and macrophage markers.** Relationship between F12 expression and M1 macrophage markers (**A**) IRF5 and (**B**) NOS2. Relationship between F12 expression and M2 macrophage markers (**C**) MS4A4A and (**D**) TGFB1.

Further, analysis using GDC TCGA data showed that the upregulated expression of F12 significantly correlated with the M1 macrophage marker IRF5 expression (P =8.2e-4) (Figure 11A), but was not significantly related to M1 macrophage marker NOS2 and M2 macrophage marker MS4A4A (all P >0.05) (Figure 11B, 11C). However, a significant positive relationship was observed between F12 expression and M2 macrophage marker TGFB1 expression (P =8.1e-7) (Figure 11D). The above findings indicated that F12 expression was correlated with immune cell infiltration levels and might promote macrophages polarization.

## DISCUSSION

F12 serves as a plasma serine protease when autoactivated into F12a upon exposure to plasma kallikrein and negatively charged surfaces [16]. F12 is involved in human atherosclerotic lesions and F12 serum levels were upregulated in early atherosclerosis in low-density lipoprotein [28]. It has also been demonstrated that F12 was related to the risk of coronary heart disease, diabetes, and sepsis [29–32]. Additionally, the elevated expression level of F12 was found in the peritoneum of epithelial ovarian cancer and the interaction of F12 and macrophages lead to cancer invasion and metastasis [33], however, it has not been extensively studied in various tumors. In this study, we identified the clinical significance of F12 in tumors and further identified its independent prognostic function in PTC. Moreover, F12 expression was associated with immune cell infiltrates especially macrophages. Our study identified F12 as a potential biomarker for predicting the prognosis of PTC patients.

Firstly, we investigated the clinical significance of F12 in pan-cancer and found that F12 was highly expressed in most cancers including BLCA, BRCA, COAD, ESCA, HNSC, KIRC, KIRP, LUAD, LUSC, PRAD, READ, SKCM, STAD, THCA, and UCEC. F12 expression was closely related to the prognosis of many cancers, indicating it has essential clinical significance in various tumors. We focused on the role of F12 in PTC in the following research.

We analyzed the F12 alteration frequency, copy number, and mutation in PTC, which indicated the overexpression of F12 in PTC. Using TCGA data, both unpaired and paired t-tests confirmed the upregulation of F12 mRNA expression in PTC. And the immunohistochemical staining results presented a higher F12 protein level in PTC tissue compared with the normal thyroid tissue, suggesting that F12 might be associated with carcinogenesis. Besides, ROC curves showed that F12 expression had high sensitivity and specificity with AUC values close to 1, indicating that F12 had a favorable diagnostic value in distinguishing PTC patients from healthy subjects. Besides, F12 overexpression contributed to the poor OS in PTC patients and Cox regression analysis exhibited F12 as an independent prognostic factor for predicting OS. These results indicated that F12 might be a robust diagnostic and prognostic biomarker in PTC. F12a was reported to initiate the intrinsic coagulation pathway, thereby contributing to fibrin clot formation [34]. The vascular endothelial growth factor (VEGF) expressed by the tumor cells induces fibrin deposition, which served as a provisional matrix for migration of inflammatory and endothelial cells, and stromal fibroblasts into the healing wound [35–38]. On the other hand, fibrin deposited in adjacent tumor vasculature and tumor macrophages with tissue factors would initiate the coagulation pathways in a vicious cycle [39, 40]. Importantly, fibrin has been reported to promote cancer cell growth and migration [41]. Besides, fibrin matrices were essential in tumor angiogenesis [42, 43]. Therefore, the authors speculated that F12 might affect the progression of PTC patients by triggering the coagulation pathway. The complement system activated by F12a is the primary immune response system in the blood circulation system, participating in the innate immune response [44]. The innate immune system exerts a major influence on the occurrence and proliferation of cancers, having an impact on the survival of cancer patients [45, 46]. This might be another explanation for the unfavorable prognosis of PTC patients with high F12 expression.

To explore the underlying mechanism of F12 involved in PTC, the KEGG pathway analysis of DEGs between the high- and low- F12 expression groups was performed. These DEGs were mainly enriched in oxidative phosphorylation, thyroid hormone synthesis, antigen processing and presentation, glutathione metabolism, and ferroptosis. The GSEA result showed that glyceropholipid metabolism, RNA polymerase, tyrosine metabolism, glycerolipid metabolism, and peroxisome were the major significant pathways in high F12 expression phenotype. Glutamine metabolism is able to inhibit glutathione synthesis and induce ferroptosis. The inhibition of glutamine metabolism can suppress ferroptosis [47]. Moreover, ferroptosis represents a crucial role in the development and treatment of various cancers, which can be both stimulatory and inhibitory. Ferroptosis induces inflammation-related immunosuppressive tumor microenvironment to facilitate tumor growth; however, ferroptosis inducers including Erastin cause ferroptosis, inhibiting the progression of tumor [48–51]. Therefore, F12 might affect the progression of PTC through metabolic pathways, especially glutamine metabolism.

Tumor microenvironment immune cells constitute a key factor of tumor tissues and exhibit a significant role in predicting the survival status and therapeutic efficacy of cancer patients [52–54]. M2 macrophages promote tumor growth through the promotion of angiogenesis, secretion of growth factors, and suppression of adaptive immunity [55, 56]. Whereas, M1 macrophages stimulate the immune response via secreting proinflammatory cytokines [57]. By analyzing the relationship between F12 expression and immune cell infiltration, we found that the fractions of many immune cells in the high F12 expression group vary from the low F12 expression group. Interestingly, the M2 macrophage infiltration level was notably higher in the high F12 expression group, while a downregulated M1 macrophage infiltration level was observed in this group. This provided new insight into the correlation between F12 overexpression and shorter OS time of PTC patients. However, the data we analyzed were obtained from the public datasets, which requires further validation by *in vitro* and *in vivo* experiments.

In summary, F12 expression had high diagnostic efficacy and its overexpression served as an independent prognostic predictor for PTC patients. In addition, F12 expression was involved in metabolic pathways especially glutathione metabolism in PTC. Our findings also suggested that F12 might play a vital role in immune cell infiltration.

### Availability of data and materials

The datasets used and/or analyzed during the current study are available from the corresponding author on reasonable request.
